# Social isolation and loneliness and their association with all-cause mortality. A population-based longitudinal study in Norway: The Tromsø Study 1994–2023

**DOI:** 10.1016/j.pmedr.2024.102930

**Published:** 2024-11-20

**Authors:** Ola Løvsletten, Tormod Brenn

**Affiliations:** Department of Community Medicine, UiT - The Arctic University of Norway, Tromsø, Norway

**Keywords:** Social isolation, Loneliness, All-cause mortality, Longitudinal study, Time-varying Cox, Tromsø Study

## Abstract

**Objectives:**

Loneliness and social isolation are associated with increased mortality, but few studies have assessed this association over long time in young adults.

**Methods:**

The study sample comprised 9061 women and 8735 men aged 25 to 69 years who participated in the Tromsø4 survey (1994–95, baseline) of the Tromsø Study, Norway. A subset of the study sample also attended the Tromsø5 (2001), Tromsø6 (2007–08), and Tromsø7 (2015–16) surveys. Participants were followed up for all-cause mortality until November 2023; with 1630 women and 2099 men deceased. Information on social isolation (least isolated, modestly isolated, and most isolated) and loneliness (yes, no) were taken from self-administered questionnaires. Sex-specific, time-varying Cox models were employed, updating exposures and covariates from Tromsø5.

**Results:**

Most-isolated versus least-isolated women and men had hazard ratios of 1.37 (95 % confidence interval 1.18–1.59) and 1.41 (1.25–1.60), respectively, after adjustment for covariates. These hazard ratios were higher in younger adults (HR = 1.55 in women and HR = 1.76 in men aged <50 years at baseline), though the age-isolation interaction was not statistically significant in women (*P* = 0.26), but in men (*P* = 0.01). For loneliness, the adjusted hazard ratios were 1.51 (1.23–1.87) and 1.46 (1.16–1.84). Over time, 51 % and 47 % of participants remained most isolated at Tromsø5 and Tromsø7, respectively; 25 % of those initially lonely remained so at Tromsø5, while only 2.6 % of those initially non-lonely became lonely at Tromsø5.

**Conclusion:**

Both social isolation and loneliness are strongly associated with all-cause mortality, particularly among younger adults, underscoring their importance as public health concerns.

## Highlights

• Social isolation and loneliness were strongly associated with an increased mortality risk.
• These associations did not vanish after adjusting for risk factors such as smoking.
• Younger adults had a higher relative risk of early death associated with social isolation.
• Social isolation was persistent over time.

## 1 Introduction

Over the years, many research papers have established risk factors for health, such as smoking, physical inactivity, alcohol consumption, high cholesterol, high blood pressure, and obesity. The role of social isolation and loneliness in health have been undervalued (Kanbay et al., 2023), but robust evidence shows that both are associated with an increased risk of premature death (Cene et al., 2022; Holt-Lunstad, 2024).

However, few studies have included young adults (aged 25–49 years at baseline). In a meta-analysis from 2015, 89 % of the studies included adults older than 50 years of age (Holt-Lunstad et al., 2015), while in a more recent meta-analysis the same number was 70 %. One study found an association between loneliness and all-cause mortality for 18 to 59-year-olds, but not for those in older age groups (Lara et al., 2020). Most of the studies have relied on baseline information and did not consider time-varying exposures or covariates. Since the experience of loneliness may change over time, it may potentially be important to incorporate time-varying exposures and covariates into models (Yu et al., 2023). Indeed, using a time-varying Cox model, MacNeil-Vromen et al. reported a null finding for perceived social support among elderly participants (>65 years at baseline) (MacNeil-Vroomen et al., 2018).

The importance of increasing the knowledge on this topic is underlined by statistics on the number of single-person households without children, which increased by 30.7 % in the European Union from 2009 to 2022 (Eurostat, 2023). The global pandemic may also have reduced our social connections (Holt-Lunstad, 2021).

Loneliness is a subjective feeling, while social isolation is an objective measure on social connections. Loneliness is often assessed with a single question, although the formulation of these questions and the response options offered vary by study. Social isolation is typically constructed from three or four questions on number of housemates, participation in various activities, and number of friends. These responses are then summed to construct a social isolation score, such as the Social Network Index (SNI, the most frequent measure seen in the literature), the Social Isolation Index, the Social Isolation Scale, the Social Integration Index, and the Social Isolation Score, among others (Wang et al., 2023). Although social isolation is complex, and a universal measure has so far not been established, constructed scores offer the possibility to categorize social isolation from least to most isolated, with one or two intermediate isolation levels (Laugesen et al., 2018).

There is an ongoing discussion on how social isolation and loneliness interact (Stokes et al., 2021), and thereby the need to distinguish between these distinct constructs (Beller and Wagner, 2018; Coyle and Dugan, 2012; Perissinotto and Covinsky, 2014). For instance, in a bad marriage, one might feel lonely despite frequent social contact. On the other hand, someone may be socially isolated without feeling lonely.

Several reasons as to why social isolation and loneliness have an adverse effect on mortality have been proposed (Cene et al., 2022; Holt-Lunstad, 2024). Both conditions are associated with high-risk behaviors such as smoking, alcohol consumption, and physical inactivity, as well as high body mass index (BMI), high lipids, and high blood pressure. It is thus necessary to study the effect of social isolation and loneliness separately, and in relation to relevant covariates (Holt-Lunstad et al., 2015).

The aim of this study was to estimate the relative risk of premature death associated with social isolation and loneliness over a 29-year follow up period, using time-varying Cox models. We also assessed whether age (25–49 versus 50–69 years at baseline) modified the risk associated with social isolation, and examined the stability of the exposures over time.

## 2 Methods

### 2.1 Study sample

This analysis used data from the Tromsø Study, an ongoing population-based study in Norway that consists of seven health surveys (Tromsø1-Tromsø7). Inhabitants of the Tromsø municipality are invited to the surveys. In Tromsø4 (1994–95) and Tromsø7 (2015–16), all those aged 25 years or more and 40 years or more, respectively, were invited; in Tromsø5 (2001) and Tromsø6 (2007–08) representative samples were invited (Eggen et al., 2013; Hopstock et al., 2022; Jacobsen et al., 2012). We used Tromsø4 as a baseline; it consisted of 12,865 men and 14,293 women between 25 and 97 years of age (Jacobsen et al., 2012). We then excluded participants who withdrew their consent (*N* = 280), those aged 70 years or more (*N* = 2752), and those with missing values on one or more of the covariates included (*N* = 6330), resulting in a final study sample of 9061 women and 8735 men aged 25–69 years (Supplementary Fig. 1). A subset of these participants attended Tromsø5 (*N* = 4724), Tromsø6 (*N* = 7722), and Tromsø7 (*N* = 9988).

### 2.2 Ethics

The Regional Committee of Research Ethics (REK) and the Data- and Publication Committee (DPC) of the Tromsø Study both approved this study (reference number REK: 578088), and the Norwegian Agency for Shared Services in Education and Research assessed this project (reference number 273247). The study followed the guidelines from the DPC.

### 2.3 Exposures

We assigned scores of 0 (= no) and 1 (= yes) to each of three domains: living with a partner/spouse, normally taking part in organized gatherings (1–2 times a month or more frequent), and having friends to talk with and give support. The responses to these domains were summed to determine social isolation (most isolated: sum = 0 or 1, modestly isolated: sum = 2, and least isolated: sum = 3). Questions in the friend domain were different in Tromsø6 and Tromsø7 than at baseline and Tromsø5, though the content was similar (Supplementary Table 1).

Loneliness was assessed by the question “Have you in the last two weeks felt lonely?” and dichotomized as not lonely (no, a little) and lonely (a lot, very much). This question was included at baseline and Tromsø5, but not Tromsø6 and Tromsø7.

### 2.4 Covariates

In the adjusted analyses, we controlled for the yes/no variables daily smoking, physical inactivity, low education, heavy alcohol consumption, high cholesterol, high blood pressure, and obesity. These covariates were chosen as they are known to be strongly linked with mortality, and possibly also social isolation and loneliness (Pantell et al., 2013; Tanskanen and Anttila, 2016; Terry et al., 2005; Collaborators, 2024).

Daily smoking at baseline was measured by the three questions: “Do you yourself smoke: Cigarettes daily? Cigars/cigarillos daily? A pipe daily?”. If participants replied yes to any of these, they were categorized as daily smokers. In Tromsø5-Tromsø7 we categorized participants as daily smokers if they replied. “Yes, now” to the question “Do you/did you smoke daily?”

Physical inactivity was determined by the question: “How has your physical activity in leisure time been during this last year? Think of your weekly average for the year. Time spent going to work counts as leisure time.”, for “light activity” (not sweating/out of breath) and “heavy activity” (sweating/out of breath). Response options were scored (none = 1; <1 h/wk. = 2; 1–2 h/wk. = 3; >3 h/wk. = 4), response categories were summed, and participants were classified as physically inactive if the sum was less than three.

Education was defined as low if the participant indicated an education level of primary/partly secondary education (up to 9 years of schooling). Heavy alcohol consumption was defined as drinking at least five small bottles of beer, a bottle of wine, or ¼ bottle of spirits approximately 1–2 times a month or more frequently.

High cholesterol was defined as total cholesterol >7 mmol/L; high blood pressure as systolic blood pressure ≥ 140 mmHg, diastolic pressure ≥ 90 mmHg or used blood pressure medication; and obesity as a BMI (=weight/height^2^) ≥30 kg/m^2^.

Low education and heavy alcohol consumption were not updated after baseline, and physical inactivity was not updated after Tromsø5, while information for the remaining covariates was updated at all surveys wherever this is indicated.

Previous studies have pointed out the need to adjust for initial health status (Holt-Lunstad et al., 2015). Therefore, we conducted a sub-analysis among participants with self-reported good health at baseline. Good health was determined by the question: “What is your current state of health?”, with response options poor, not so good, good, and very good. Those who answered good or very good were considered to have good health.

### 2.5 Follow-up

Follow-up for emigration and death was performed through linkage to the Population Register of Norway, using the national, unique 11-digit personal identification number. Date of birth, survey attendance, emigration from Norway, and all-cause death were recorded. End of follow-up was defined as age at emigration, death, or end of follow-up (27 November 2023), whichever came first.

### 2.6 Statistical analysis

Kaplan-Meier plots were used to display survival curves according to social isolation and loneliness. Time-varying Cox models, providing hazard ratios (HRs) with 95 % confidence intervals (CIs), were used to assess the effect of social isolation and loneliness in univariate and multiple analyses. We used age as a time scale, stratified by 5-year birth cohort (Canchola et al., 2003; Korn et al., 1997; Vyas et al., 2021). Residual plots indicated that the proportional hazards assumption was not violated. We conducted sex-specific analyses to account for possible differences in the associations between women and men (Laugesen et al., 2018).

We included data from baseline and Tromsø5 in the main analysis, as well as in a sub-analysis of those with self-reported good health at baseline and those under 50 years of age. To test if the effect differed between younger and older adults, we included interaction terms between the exposures and an indicator variable for those under 50 years of age.

By delaying the exposure by a given time, one may check for reverse causality (Therneau et al., 2017). Thus, we performed a sensitivity analysis in which we delayed the exposures by two years and excluded those who died less than two years after baseline. As a robustness test, we repeated this analysis using time-varying Cox models, in which we updated data on all available exposures and covariates at all four surveys for those under 77 years of age at the time of the survey. Finally, we assessed the stability of the exposures by simple cross-tabulation between surveys.

We required complete observations at baseline, which may bias the results. To assess this potential bias, we compared Cox models with multiple imputed datasets to the complete observation case. For the multiple imputation we used the R package *smcfcs (package version 1.8.0),* which uses the method described in ref*. (*Bartlett et al., *2015**)*. Here we used time since baseline as time scale due to requirements of this package, and included age as a covariate. The bias in the hazard ratios were 0.1 or less in the unadjusted models and 0.05 or less in the adjusted models (Supplementary Table 6).

The analyses was performed with Rstudio version 4.2.2 (R Core Team, 2022).

## 3 Results

### 3.1 Baseline

At baseline, 40 % of women and 33 % of men were classified as least isolated, 47 % and 49 % as moderately isolated, and 13 % and 18 % as most isolated. Loneliness was much less prevalent, with only 2.7 % of women and 2.3 % of men reporting feeling lonely the last 14 days. Social isolation was strongly correlated with loneliness (Table 1). For instance, the prevalence of loneliness was 1.0 %, 2.6 %, and 8.8 % among the least-, moderately-, and most-isolated women, respectively.

**Table 1:** Baseline characteristics by social isolation^a^ and sex among Norwegian adults aged 25–69 years at baseline (Tromsø4, 1994–95). The Tromsø Study.

	Women				Men			
Characteristic	Least *N* = 3608	Modestly *N* = 4277	Most *N* = 1176	P-value^b^	Least *N* = 2883	Modestly *N* = 4279	Most *N* = 1573	P-value^b^
Loneliness^c^, %	1.0	2.6	8.8	<0.01	0.3	1.8	7.5	<0.01
Age (years), mean	43.4	42.8	43.3	0.02	43.3	43.8	44.0	0.22
Daily smoking, %	30.6	40.9	49.1	<0.01	28.9	38.4	46.3	<0.01
Physical inactivity, %	12.3	17.4	20.1	<0.01	9.5	16.8	21.9	<0.01
Low education, %	25.2	30.3	30.7	<0.01	16.9	26.3	31.2	<0.01
Heavy alcohol consumption, %	11.5	13.2	20.1	<0.01	33.0	37.2	41.6	<0.01
High cholesterol, %	16.1	18.0	18.5	0.04	17.7	19.9	20.7	0.02
High blood pressure, %	21.4	22.0	25.0	0.03	32.8	38.1	38.0	<0.01
Obesity, %	8.8	9.0	10.6	0.16	8.0	9.5	10.7	<0.01
Self-reported good health, %	75.7	73.0	69.2	<0.01	84.6	79.1	71.3	<0.01

a Social isolation constructed from i) Living with a partner/spouse ii) participating in organized gatherings monthly or more frequent and iii) having friends to talk with and give support. Least isolated: Yes on all three (i-iii); modestly isolated: Yes on two out of the three; most isolated: Yes on zero or one of the three.

b Pearson's Chi-squared test; Kruskal-Wallis rank sum test.

c “Have you in the last two weeks felt lonely?” dichotomized to not lonely (no, a little) and lonely (a lot, very much).

The prevalence of investigated covariates, such as daily smoking and physical inactivity, were highest among the most-isolated and lowest among the least-isolated participants, with modestly-isolated participants falling in between – except for high blood pressure in modestly- and most-isolated men. Similarly, lonely participants had a worse risk profile than those who were not lonely (Supplementary Table 2).

**Fig. 1:**
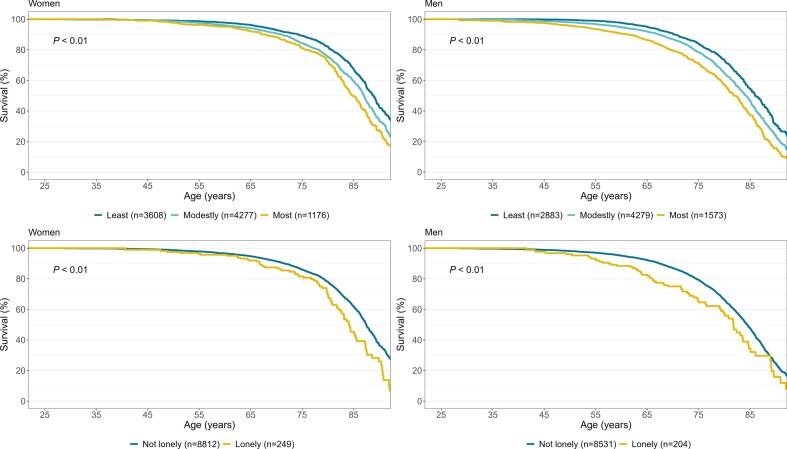
Survival curves, with *P*-values of the log-rank test, according to baseline social isolation^a^ (least isolated, modestly isolated, most isolated) and loneliness^b^ among Norwegian adults aged 25–69 years at baseline (Tromsø4, 1994–95). The Tromsø Study, 1994–2023. ^a^ Social isolation constructed from i) Living with a partner/spouse ii) participating in organized gatherings monthly or more frequent and iii) having friends to talk with and give support. Least isolated: Yes on all three (i-iii); modestly isolated: Yes on two out of the three; most isolated: Yes on zero or one of the three. ^b^ “Have you in the last two weeks felt lonely?” dichotomized to not lonely (no, a little) and lonely (a lot, very much).

In Fig. 1, participants enter the analysis at age 25 to 69 years and are followed for up to 29 years. Thus, the youngest enter the analysis at the left-most part of the figure. The survival curves started to differentiate at age 40 to 50 years. Survival was highest among those least isolated and lowest for the most isolated, with modestly-isolated participants falling in between. Moreover, survival was lower among lonely participants compared to those who were not lonely (Fig. 1).

### 3.2 Main analysis

Fig. 1 only used baseline information and did not include covariates. We addressed this issue by considering possible time-varying exposures and covariates. For those who were alive seven years after baseline and attended Tromsø5, we updated the exposures and covariates in the Cox models. The HRs were lower in the adjusted models when compared to the unadjusted models. The HRs in the joint analysis were similar to those observed when the exposures were analyzed separately (Table 2). For instance, the most-isolated women had a 1.68 higher hazard than the least-isolated women. However, when adjusted for covariates, such as daily smoking and physical inactivity, the HR dropped to 1.37, and to 1.32 when loneliness was included in the model. We note that the CIs for loneliness were wide, which reflects the low number of lonely people. Finally, except for daily smoking, the HRs were of similar, or even greater magnitude than most of the covariate HRs (Supplementary Table 3).

**Table 2:** Hazard ratios (HRs) with 95 % confidence intervals (CIs) of social isolation and loneliness in Norwegian adults aged 25–69 years at baseline^a^(Tromsø4, 1994–95). The Tromsø Study 1994–2023.

	Women *N* = 9061, Deaths = 1630				
	Unadjusted		Adjusted^b^		Joint, adjusted^b^
Characteristic	HR (95 % CI)	HR (95 % CI)	HR (95 % CI)	HR (95 % CI)	HR (95 % CI)
Social isolation^c^					
Least isolated		1 (reference)		1 (reference)	1 (reference)
Modestly isolated		1.32 (1.18–1.47)		1.20 (1.07–1.34)	1.20 (1.07–1.34)
Most isolated		1.68 (1.46–1.94)		1.37 (1.18–1.59)	1.32 (1.14–1.53)
Loneliness^d^	1.77 (1.44–2.19)		1.51 (1.23–1.87)		1.43 (1.16–1.78)


	Men *N* = 8735, Deaths = 2099				
	Unadjusted		Adjusted^b^		Joint, adjusted^b^
Characteristic	HR (95 % CI)	HR (95 % CI)	HR (95 % CI)	HR (95 % CI)	HR (95 % CI)
Social isolation^c^					
Least isolated		1 (reference)		1 (reference)	1 (reference)
Modestly isolated		1.24 (1.12–1.37)		1.10 (0.99–1.22)	1.10 (0.99–1.22)
Most isolated		1.70 (1.50–1.92)		1.41 (1.25–1.60)	1.39 (1.22–1.58)
Loneliness^d^	1.55 (1.23–1.95)		1.46 (1.16–1.84)		1.35 (1.07–1.70)

a Exposures and covariates were updated with data from Tromsø5 (2001) when available.

b Daily smoking, physical inactivity, low education, heavy alcohol consumption, high cholesterol, high blood pressure, and obesity.

c Social isolation constructed from i) Living with a partner/spouse ii) participating in organized gatherings monthly or more frequent and iii) having friends to talk with and give support. Least isolated: Yes on all three (i-iii); modestly isolated: Yes on two out of the three; most isolated: Yes on zero or one of the three.

d “Have you in the last two weeks felt lonely?” dichotomized to not lonely (no, a little) and lonely (a lot, very much). Not lonely reference.

### 3.3 Sub-analysis

HRs for social isolation in the sub-analysis of participants with self-reported good health at baseline were of similar magnitude as in the main analysis (Table 3). In a sub-analysis restricted to those younger than 50 years at baseline (Table 4), a higher HR was observed among the most-isolated men (HR = 1.76 compared to HR = 1.41 in the main analysis). A test for interaction indicated that age was an effect-modifier for social isolation in men (*P* = 0.01 for most isolated), but not in women (*P* = 0.26).

**Table 3:** Hazard ratios (HRs) with 95 % confidence intervals (CIs) of social isolation and loneliness among Norwegian adults, aged 25–69 years with self-reported good health at baseline^a^ (Tromsø4, 1994–95). The Tromsø Study, 1994–2023.

	Women *N* = 6663, Deaths = 862				
	Unadjusted		Adjusted^b^		Joint, adjusted^b^
Characteristic	HR (95 %CI)	HR (95 %CI)	HR (95 %CI)	HR (95 %CI)	HR (95 %CI)
Social isolation^c^					
Least isolated		1 (reference)		1 (reference)	1 (reference)
Modestly isolated		1.30 (1.12–1.51)		1.19 (1.02–1.38)	1.19 (1.02–1.38)
Most isolated		1.70 (1.39–2.06)		1.44 (1.18–1.76)	1.42 (1.16–1.74)
Loneliness^d^	1.42 (0.95–2.12)		1.34 (0.90–2.00)		1.23 (0.82–1.85)


	Men *N* = 6940, Deaths = 1324				
	Unadjusted		Adjusted^b^		Joint, adjusted^b^
Characteristic	HR (95 % CI)	HR (95 % CI)	HR (95 % CI)	HR (95 % CI)	HR (95 % CI)
Social isolation^c^					
Least isolated		1 (reference)		1 (reference)	1 (reference)
Modestly isolated		1.27 (1.12–1.44)		1.11 (0.98–1.26)	1.11 (0.98–1.27)
Most isolated		1.76 (1.51–2.06)		1.49 (1.27–1.75)	1.49 (1.27–1.75)
Loneliness^d^	1.24 (0.85–1.82)		1.21 (0.83–1.77)		1.07 (0.73–1.58)

a Exposures and covariates were updated with data from Tromsø5 (2001) when available.

b Daily smoking, physical inactivity, low education, heavy alcohol consumption, high cholesterol, high blood pressure, and obesity.

c Social isolation constructed from i) Living with a partner/spouse ii) participating in organized gatherings monthly or more frequent and iii) having friends to talk with and give support. Least isolated: Yes on all three (i-iii); modestly isolated: Yes on two out of the three; most isolated: Yes on zero or one of the three.

d “Have you in the last two weeks felt lonely?” dichotomized to not lonely (no, a little) and lonely (a lot, very much). Not lonely reference.

**Table 4:** Hazard ratios (HRs) with 95 % confidence intervals (CIs) of social isolation and loneliness among Norwegian adults aged 25–49 years at baseline^a^ (Tromsø4, 1994–95). The Tromsø Study, 1994–2023.

	Women *N* = 6612, Deaths = 440				
	Unadjusted		Adjusted^b^		Joint, adjusted^b^
Characteristic	HR (95 %CI)	HR (95 %CI)	HR (95 %CI)	HR (95 %CI)	HR (95 %CI)
Social isolation^c^					
Least isolated		1 (reference)		1 (reference)	1 (reference)
Modestly isolated		1.29 (1.05–1.59)		1.16 (0.94–1.44)	1.16 (0.94–1.43)
Most isolated		1.84 (1.40–2.44)		1.55 (1.17–2.06)	1.51 (1.14–2.02)
Loneliness^d^	1.75 (1.09–2.81)		1.50 (0.93–2.42)		1.35 (0.83–2.18)


	Men *N* = 6211, Deaths = 623				
	Unadjusted		Adjusted^b^		Joint, adjusted^b^
Characteristic	HR (95 % CI)	HR (95 % CI)	HR (95 % CI)	HR (95 % CI)	HR (95 % CI)
Social isolation^c^					
Least isolated		1 (reference)		1 (reference)	1 (reference)
Modestly isolated		1.47 (1.22–1.78)		1.27 (1.05–1.55)	1.27 (1.04–1.55)
Most isolated		2.21 (1.77–2.77)		1.76 (1.40–2.22)	1.73 (1.37–2.19)
Loneliness^d^	1.64 (1.06–2.54)		1.50 (0.97–2.32)		1.31 (0.84–2.03)

a Exposures and covariates were updated with data from Tromsø5 (2001) when available.

b Daily smoking, physical inactivity, low education, heavy alcohol consumption, high cholesterol, high blood pressure, and obesity.

c Social isolation constructed from i) Living with a partner/spouse ii) participating in organized gatherings monthly or more frequent and iii) having friends to talk with and give support. Least isolated: Yes on all three (i-iii); modestly isolated: Yes on two out of the three; most isolated: Yes on zero or one of the three.

d “Have you in the last two weeks felt lonely?” dichotomized to not lonely (no, a little) and lonely (a lot, very much). Not lonely reference.

### 3.4 Sensitivity analysis

Results from our sensitivity analysis in which exposures were delayed by two years were not substantially different from the main analysis (Supplementary Table 4), even after updating the exposures and covariates at all four surveys (Supplementary Table 5).

Social isolation was persistent, i.e., positively correlated, over time, with 66 % and 52 % of participants remaining in the same category at seven and twenty-one years of follow-up, respectively. Only 1.8 % and 5.6 % of participants moved from least to most isolated or most to least isolated, at seven and twenty-one years of follow-up. Among those most isolated at baseline, 50.8 % and 47.4 % remained most isolated at seven and twenty-one years of follow-up, respectively. Twenty-five percent of those who were lonely at baseline stayed lonely, whereas only 2.6 % of those who were not lonely became lonely at seven years of follow-up (Table 5).

**Table 5:** Cross-tabulation (N (row-%)) of social isolation and loneliness at baseline (Tromsø4, 1994–95) and at Tromsø5 (2001); and of social isolation at Tromsø7 (2015–16), among Norwegian adults aged 25–69 years in Tromsø4. The Tromsø Study, 1994–2016.

	Tromsø5 (2001)^a^			Tromsø7 (2015–16)^b^		
Baseline (Tromsø4, 1994–95)	Least isolated	Modestly isolated	Most isolated	Least isolated	Modestly isolated	Most isolated
Social isolation^c^						
Least isolated	866 (68.1)	374 (29.4)	31 (2.4)	1917 (50.0)	1507 (39.3)	413 (10.8)
Modestly isolated	347 (22.1)	1078 (68.7)	144 (9.2)	1018 (23.3)	2374 (54.3)	978 (22.4)
Most isolated	29 (6.6)	186 (42.6)	222 (50.8)	112 (10.2)	464 (42.4)	519 (47.4)
	Not lonely	Lonely				
Loneliness^d^						
Not lonely	4042 (97.4)	108 (2.6)				
Lonely	84 (75.0)	28 (25.0)				

a The percentages of identical answers were 66.1 and 95.5 for social isolation and loneliness, respectively.

b The percentage of identical answers was 51.7.

c Social isolation constructed from i) Living with a partner/spouse ii) participating in organized gatherings monthly or more frequent and iii) having friends to talk with and give support. Least isolated: Yes on all three (i-iii); modestly isolated: Yes on two out of the three; most isolated: Yes on zero or one of the three.

d “Have you in the last two weeks felt lonely?” dichotomized to not lonely (no, a little) and lonely (a lot, very much).

## 4 Discussion

We have presented analyses of how social isolation and loneliness relate to all-cause mortality in a large population of women and men aged 25 to 69 years at baseline, followed up for 29 years. The main finding is the strong association of both social isolation and loneliness with all-cause mortality. Our results indicate that, in men in particular, but also in women, the relative risk of premature mortality was higher for younger adults. The main finding can partly be explained by a considerably worse risk profile among those exposed to social isolation or loneliness. However, even after adjusting for covariates such as daily smoking and high blood pressure, we observed a significant, independent association for both exposures. These results applied both to the general population and to a sub-sample of individuals who were healthy at baseline.

The strong association between social isolation, loneliness, and mortality have been reported in numerous previous studies (Holt-Lunstad et al., 2015; Wang et al., 2023; Alcaraz et al., 2019; Rico-Uribe et al., 2018; Yu et al., 2022; Zhou et al., 2023). However, most previous research on social isolation has been done on older adults. Interestingly, we observed that young age (25–49 years old at baseline) amplified the relative risk of premature mortality associated with social isolation. We also observed that the survival curves started to differentiate at age 40 to 50 years (Fig. 1).

Previous studies have primarily relied on baseline measurements of social isolation and loneliness. Interestingly, we found that using time-varying values did not alter these associations much. Our findings, at least for men, contrast with the null findings in MacNeil-Vroomen et al (MacNeil-Vroomen et al., 2018). One possible explanation for this discrepancy is the different questions used in that study. Another possible explanation is age-related, i.e., that loneliness is more important at young and middle age than later in life. However, using *cumulative* loneliness measures, Yu et al also found a strong association with mortality among middle-aged and older adults (Yu et al., 2023).

Social isolation has been described to have an equivalent or even greater influence on mortality risk than traditional behavioral and clinical risk factors (Holt-Lunstad et al., 2015; Pantell et al., 2013). Except for daily smoking, which had a higher HR, our findings are consistent with this description. Our findings also suggest a dose-response relationship, with moderately-isolated individuals having a mortality risk between that of our least- and most-isolated participants. Similar findings have been reported in other large population-based studies (Laugesen et al., 2018; Pantell et al., 2013; Tanskanen and Anttila, 2016). There is probably also a threshold effect, with only isolation beyond a certain level being harmful to one's health. Findings from the UK Biobank indicated that this threshold for visits from family and friends is about once a month (Foster et al., 2023).

For loneliness, the increased mortality risks of 1.51 and 1.46 in women and men, respectively, are higher than the 1.14 reported in a recent systematic review and meta-analysis (Wang et al., 2023), though it should be noted that the CIs for loneliness were wide in this study. For social isolation, the same paper presented a risk of 1.32, which corresponds with the 1.33 value reported in another recent review and meta-analysis (Naito et al., 2023). We found HRs of 1.37 in women and 1.41 in men, which are close to these meta-analyses. However, results vary from study to study, due to the different questions used, length of follow-up, covariates included, and perhaps also geographical location. Other Nordic studies have reported higher social isolation values than ours, with rates of 2.49 from Finland (Tanskanen and Anttila, 2016), 1.7 in men and 1.6 in women of Denmark (Laugesen et al., 2018), and 2.54 for Sweden (Lennartsson et al., 2022). We note that the Finnish study did not adjust for smoking, which we found to be the most important covariate.

Impaired health is a strong predictor of mortality (Lorem et al., 2020) and may also lead to reduced social connection, such as discontinuing participation in organized activities or having less frequent contact with friends. Indeed, we observed a substantial association between self-reported health at baseline and degree of social isolation and loneliness. Thus, some of the main findings may be explained by reversed causality, i.e., social isolation and loneliness following impaired health. However, this is unlikely, since the HRs for both exposures among participants with self-reported good health at baseline were similar to those in the main analyses, thus eliminating the possibility of reverse causality. Delaying the exposure by two years as we did also reduced the chance of reverse causality.

### 4.1 Strengths and limitations

Our findings extend the literature by using data from a high-quality, large-scale, population-based study. Strengths of the Tromsø Study include anthropometric measurements, blood measurements, and high attendance; 65 % to 79 % in the four surveys used in this study. We consider the comprehensive analysis to be a substantial strength of this study and highlight that we assessed the stability of the exposures and tried to utilize available repeated measurements. The long follow-up time is also a strength since this increase the statistical power. This may be particularly important among the young adults.

Those most lonely and most socially isolated are probably less likely to attend the surveys, and this may bias the results towards the null. Indeed, attendance was lower among unmarried individuals in Tromsø7 (Vo et al., 2023). Another limitation is the relatively small number of lonely people in this study. Loneliness was assessed using a single question instead of multiple questions like those in the validated UCLA (University of California, Los Angeles*)* loneliness scale. Additionally, the single question used the word *lonely* rather than asking indirectly such as in the UCLA. The disadvantage of asking directly is that it can be perceived as stigmatizing (Barstad, 2021).

## 5 Conclusion

Social isolation, and to a lesser degree loneliness, are persistent over time and are strongly associated with all-cause mortality, even after adjusting for other risk factors. This association was observed in both the general population and in participants with self-reported good health at baseline. Thus, social isolation, and to a lesser degree, loneliness, are considerable public health problems.

## Funding

Funding: None.

## CRediT authorship contribution statement

**Ola Løvsletten:** Writing – review & editing, Writing – original draft, Visualization, Software, Methodology, Data curation, Conceptualization. **Tormod Brenn:** Writing – review & editing, Writing – original draft.

## Declaration of competing interest

The authors declare that they have no known competing financial interests or personal relationships that could have appeared to influence the work reported in this paper.

## Data Availability

The data are available upon application for data access to the Tromsø Study, see www.tromsostudy.com and www.tromsoundersokelsen.no

## Appendix A Supplementary data

Supplementary material Image 1
