# Supplementary material for: Social isolation and loneliness and their association with all-cause mortality. A population-based longitudinal study in Norway: The Tromsø Study 1994–2023
Source: Prev Med Rep. 2024 Nov 20;48:102930. doi: 10.1016/j.pmedr.2024.102930 (PMC11621603; doi:10.1016/j.pmedr.2024.102930)
Supplement: Supplementary file 1 — Supplementary material [file mmc1.docx]

**Supplementary material to** ***Social isolation and loneliness and their association with all-cause mortality. A population-based longitudinal study* in Norway*: The Tromsø Study 1994-2023.***

Ola Løvsletten and Tormod Brenn.

**Social isolation**

The social isolation construct was based on three components of a self-administered questionnaire: partnership, associations, and friends. Supplementary Table 1 displays the variable names, questions with response alternatives, and in which of the surveys these variables were included.

Exposure to social isolation was computed as the sum of responses to the three components each contributing with 1 or 0 (Partnership, yes = 1, no = 0; Associations, 1-2 times a month or more often = 1, else = 0; Friends, one or more = 1, else = 0 (Tromsø4 and Tromsø 5), or yes on both variables = 1, else = 0 (Tromsø6 and Tromsø 7)).

Thus the social isolation sum score ranged from 0 to 3, and we categorized participants with a score of 3 as least-isolated, 2 as moderately isolated, and 0 or 1 as most-isolated.

Supplementary Table 1. Construction of the social isolation variable. The Tromsø Study, 1994-2016.

| Tromsø Study variable name | Question | Response alternatives | Included in survey^a^ |
| --- | --- | --- | --- |
| LIVE_WITH_SPOUSE | Do you live with a spouse/partner? | Yes  No | 4,5,6, and 7 |
| ASSOCIATION | How often do you normally take part in organised gatherings, e.g. sewing circles, sports clubs, political meetings, religious or other associations? | Never, or just a few times a year  1-2 times a month  Approximately once a week  More than once a week | 4,5,6, and 7 |
| N_OF_FRIENDS | How many good friends do you have whom you can talk confidentially with and who give you help when you need it? Do not count people you live with but do include other relatives. | Number | 4 and 5 |
| SUPPORT_FRIENDS | Do you have enough friends who can give you help and support when you need it? | Yes  No | 6 and 7 |
| TALK_FRIENDS | Do you have enough friends you can talk confidentially with? | Yes  No | 6 and 7 |

^a^ 4 – Tromsø4 (1994-95), 5 – Tromsø5 (2001), 6 – Tromsø6 (2007-08), 7 – Tromsø7 (2015-16).


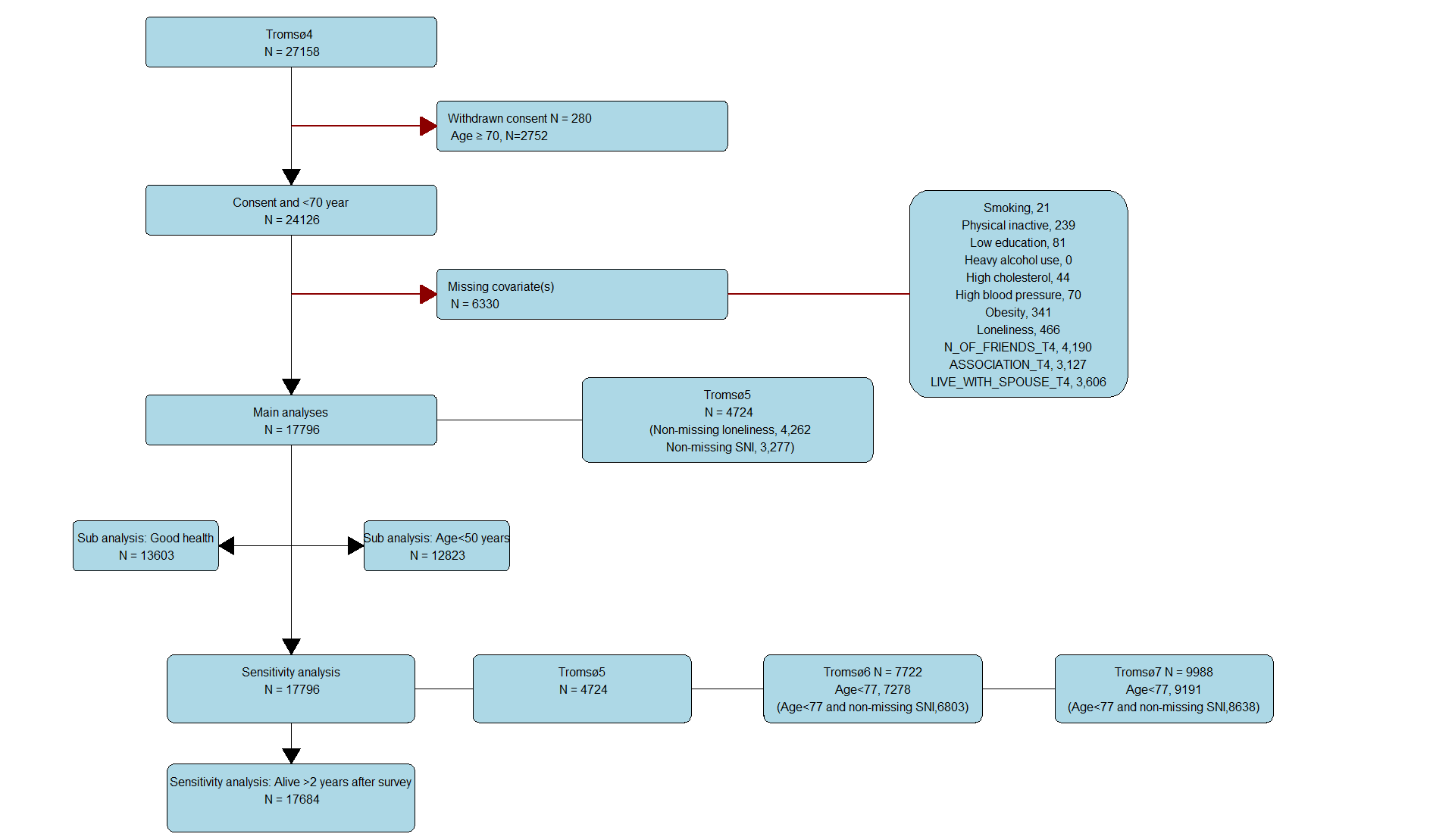

Supplementary Figure 1. Flow chart of the study sample. The Tromsø Study, 1994-2016.

Supplementary Table 2. Baseline characteristics of the study sample (Norwegian adults aged 25-69 years), by loneliness^a^ and sex. The Tromsø Study 1994-95.

|  | Women | | |  | Men | | |
| --- | --- | --- | --- | --- | --- | --- | --- |
| Characteristic | Not lonely  N=8,812 | Lonely  N=249 | P-value^c^ |  | Not lonely  N=8,531 | Lonely  N=204 | P-value^c^ |
| Social isolation^b^, % |  |  | <0.01 |  |  |  | 0.14 |
| Least isolated | 40.5 | 14.5 | <0.01 |  | 33.7 | 4.4 | <0.01 |
| Moderately isolated | 47.3 | 44.2 |  |  | 49.3 | 37.7 |  |
| Most isolated | 12.2 | 41.4 |  |  | 17.1 | 57.8 |  |
| Age (years), mean | 43.0 | 45.9 |  |  | 43.7 | 45.0 |  |
| Daily smoking, % | 37.4 | 53.8 | <0.01 |  | 36.4 | 49.5 | <0.01 |
| Physical inactivity, % | 15.4 | 27.3 | <0.01 |  | 15.2 | 20.6 | 0.03 |
| Low education, % | 28.0 | 39.0 | <0.01 |  | 23.8 | 34.3 | <0.01 |
| Heavy alcohol consumption, % | 13.3 | 16.1 | 0.21 |  | 36.5 | 42.2 | 0.1 |
| High cholesterol, % | 17.2 | 21.3 | 0.09 |  | 19.3 | 21.1 | 0.52 |
| High blood pressure, % | 22.0 | 25.7 | 0.17 |  | 36.3 | 38.2 | 0.58 |
| Obesity, % | 9.0 | 14.1 | <0.01 |  | 9.2 | 12.3 | 0.13 |
| Self-reported good health, % | 74.5 | 42.2 | <0.01 |  | 80.2 | 49.5 | <0.01 |

^a^ “Have you in the last two weeks felt lonely?” dichotomized to not lonely (no, a little) and lonely (a lot, very much).
^b^ Social isolation constructed from i) Living with a partner/spouse ii) participating in organized gatherings monthly or more frequent and iii) having friends to talk with and give support. Least isolated: Yes on all three (i-iii); modestly isolated: Yes on two out of the three; most isolated: Yes on zero or one of the three. ^c^ Pearson's Chi-squared test; Kruskal-Wallis rank sum test.

Supplementary Table 3. Hazard ratios (HRs) with 95% confidence intervals (CIs) of social isolation, loneliness, and covariates in Norwegian adults, aged 25-69-years at baseline^a^ (Tromsø4, 1994-95). The Tromsø Study, 1994-2023.

|  | Women N=9,061, Deaths=1,630 | | | | | | |
| --- | --- | --- | --- | --- | --- | --- | --- |
| Characteristic | HR (95% CI) | HR (95% CI) |  | HR (95% CI) | HR (95% CI) |  | HR (95% CI) |
| Social isolation^b^ |  |  |  |  |  |  |  |
| Least isolated |  | 1 (reference) |  |  | 1 (reference) |  | 1 (reference) |
| Modestly isolated |  | 1.32 (1.18-1.47) |  |  | 1.20 (1.07-1.34) |  | 1.20 (1.07-1.34) |
| Most isolated |  | 1.68 (1.46-1.94) |  |  | 1.37 (1.18-1.59) |  | 1.32 (1.14-1.53) |
| Loneliness^c^ | 1.77 (1.44-2.19) |  |  | 1.51 (1.23-1.87) |  |  | 1.43 (1.16-1.78) |
| Daily smoking |  |  |  | 2.05 (1.85-2.28) | 2.00 (1.79-2.22) |  | 1.98 (1.78-2.21) |
| Physical inactivity |  |  |  | 1.21 (1.07-1.36) | 1.19 (1.05-1.34) |  | 1.19 (1.05-1.34) |
| Low education |  |  |  | 1.09 (0.98-1.21) | 1.08 (0.97-1.20) |  | 1.07 (0.97-1.19) |
| Heavy alcohol consumption |  |  |  | 1.07 (0.90-1.27) | 1.06 (0.89-1.26) |  | 1.07 (0.90-1.27) |
| High cholesterol |  |  |  | 0.94 (0.85-1.05) | 0.95 (0.85-1.06) |  | 0.95 (0.85-1.06) |
| High blood pressure |  |  |  | 1.30 (1.16-1.46) | 1.29 (1.16-1.45) |  | 1.29 (1.15-1.45) |
| Obesity |  |  |  | 1.04 (0.92-1.19) | 1.04 (0.91-1.18) |  | 1.03 (0.91-1.18) |
|  | | | | | | | |
|  | Men N=8,735, Deaths=2,099 | | | | | | |
| Characteristic | HR (95 % CI) | HR (95 % CI) |  | HR (95 % CI) | HR (95 % CI) |  | HR (95 % CI) |
| Social isolation^b^ |  |  |  |  |  |  |  |
| Least isolated |  | 1 (reference) |  |  | 1 (reference) |  | 1 (reference) |
| Modestly isolated |  | 1.24 (1.12-1.37) |  |  | 1.10 (0.99-1.22) |  | 1.10 (0.99-1.22) |
| Most isolated |  | 1.70 (1.50-1.92) |  |  | 1.41 (1.25-1.60) |  | 1.39 (1.22-1.58) |
| Loneliness^c^ | 1.55 (1.23-1.95) |  |  | 1.46 (1.16-1.84) |  |  | 1.35 (1.07-1.70) |
| Daily smoking |  |  |  | 1.92 (1.76-2.11) | 1.88 (1.72-2.06) |  | 1.88 (1.72-2.06) |
| Physical inactivity |  |  |  | 1.31 (1.17-1.47) | 1.28 (1.14-1.44) |  | 1.28 (1.14-1.43) |
| Low education |  |  |  | 1.21 (1.10-1.32) | 1.18 (1.08-1.30) |  | 1.18 (1.08-1.30) |
| Heavy alcohol consumption |  |  |  | 1.24 (1.13-1.36) | 1.23 (1.12-1.35) |  | 1.23 (1.12-1.35) |
| High cholesterol |  |  |  | 1.22 (1.10-1.35) | 1.21 (1.09-1.33) |  | 1.21 (1.09-1.34) |
| High blood pressure |  |  |  | 1.36 (1.24-1.49) | 1.36 (1.24-1.49) |  | 1.36 (1.24-1.49) |
| Obesity |  |  |  | 1.30 (1.15-1.46) | 1.30 (1.15-1.46) |  | 1.30 (1.15-1.46) |

^a^ Exposures and covariates were updated with data from Tromsø5 (2001) when available.
^b^ Social isolation constructed from i) Living with a partner/spouse ii) participating in organized gatherings monthly or more frequent and iii) having friends to talk with and give support. Least isolated: Yes on all three (i-iii); modestly isolated: Yes on two out of the three; most isolated: Yes on zero or one of the three.
^c^ “Have you in the last two weeks felt lonely?” dichotomized to not lonely (no, a little) and lonely (a lot, very much). Not lonely reference.

Supplementary Table 4. Delayed exposure: Hazard ratios (HRs) with 95% confidence intervals (CIs) of social isolation and loneliness delayed by 2 years in 25-69-year-old participants at baseline^a^ (Tromsø4, 1994-95). The Tromsø Study, 1994-2023.

|  | Women N=9,061, Deaths=1,630 | | | | | | |
| --- | --- | --- | --- | --- | --- | --- | --- |
|  | Unadjusted | |  | Adjusted^b^ | |  | Joint, adjusted^b^ |
| Characteristic | HR (95% CI) | HR (95% CI) |  | HR (95% CI) | HR (95% CI) |  | HR (95% CI) |
| Social isolation^c^ |  |  |  |  |  |  |  |
| Least isolated |  | 1 (reference) |  |  | 1 (reference) |  | 1 (reference) |
| Modestly isolated |  | 1.32 (1.18-1.47) |  |  | 1.20 (1.07-1.34) |  | 1.20 (1.07-1.34) |
| Most isolated |  | 1.66 (1.46-1.94) |  |  | 1.37 (1.18-1.59) |  | 1.32 (1.14-1.53) |
| Loneliness^d^ | 1.73 (1.40-2.14) |  |  | 1.49 (1.20-1.85) |  |  | 1.41 (1.13-1.76) |
|  | | | | | | | |
|  | Men N=8,735, Deaths=2,099 | | | | | | |
|  | Unadjusted | |  | Adjusted^b^ | |  | Joint, adjusted^b^ |
| Characteristic | HR (95 % CI) | HR (95 % CI) |  | HR (95 % CI) | HR (95 % CI) |  | HR (95 % CI) |
| Social isolation^c^ |  |  |  |  |  |  |  |
| Least isolated |  | 1 (reference) |  |  | 1 (reference) |  | 1 (reference) |
| Modestly isolated |  | 1.24 (1.12-1.38) |  |  | 1.09 (0.98-1.21) |  | 1.09 (0.98-1.21) |
| Most isolated |  | 1.67 (1.47-1.89) |  |  | 1.37 (1.21-1.56) |  | 1.34 (1.18-1.53) |
| Loneliness^d^ | 1.54 (1.22-1.96) |  |  | 1.48 (1.17-1.87) |  |  | 1.38 (1.09-1.74) |

^a^ Exposures and covariates were updated with data from Tromsø5 (2001) when available.
^b^ Daily smoking, physical inactivity, low education, heavy alcohol consumption, high cholesterol, high blood pressure, and obesity.
^c^ Social isolation constructed from i) Living with a partner/spouse ii) participating in organized gatherings monthly or more frequent and iii) having friends to talk with and give support. Least isolated: Yes on all three (i-iii); modestly isolated: Yes on two out of the three; most isolated: Yes on zero or one of the three.
^d^ “Have you in the last two weeks felt lonely?” dichotomized to not lonely (no, a little) and lonely (a lot, very much). Not lonely reference.

Supplementary Table 5. Updates from 4 surveys: Hazard ratios (HRs) with 95% confidence intervals (CIs) of social isolation and loneliness in Norwegian adults, aged 25-69-years at baseline^a^ (Tromsø4, 1994-95). The Tromsø Study, 1994-2023.

|  | Women N=9,061, Deaths=1,630 | | | | | | |
| --- | --- | --- | --- | --- | --- | --- | --- |
|  | Unadjusted | |  | Adjusted^b^ | |  | Joint, adjusted^b^ |
| Characteristic | HR (95% CI) | HR (95% CI) |  | HR (95% CI) | HR (95% CI) |  | HR (95% CI) |
| Social isolation^c^ |  |  |  |  |  |  |  |
| Least isolated |  | 1 (reference) |  |  | 1 (reference) |  | 1 (reference) |
| Modestly isolated |  | 1.27 (1.13-1.42) |  |  | 1.16 (1.04-1.30) |  | 1.16 (1.04-1.30) |
| Most isolated |  | 1.58 (1.38-1.81) |  |  | 1.33 (1.16-1.53) |  | 1.29 (1.12-1.48) |
| Loneliness^d^ | 1.77 (1.44-2.19) |  |  | 1.55 (1.25-1.91) |  |  | 1.47 (1.19-1.82) |
|  | | | | | | | |
|  | Men N=8,735, Deaths=2,099 | | | | | | |
|  | Unadjusted | |  | Adjusted^b^ | |  | Joint, adjusted^b^ |
| Characteristic | HR (95 % CI) | HR (95 % CI) |  | HR (95 % CI) | HR (95 % CI) |  | HR (95 % CI) |
| Social isolation^c^ |  |  |  |  |  |  |  |
| Least isolated |  | 1 (reference) |  |  | 1 (reference) |  | 1 (reference) |
| Modestly isolated |  | 1.23 (1.11-1.37) |  |  | 1.17 (1.05-1.10) |  | 1.17 (1.05-1.30) |
| Most isolated |  | 1.53 (1.35-1.73) |  |  | 1.40 (1.24-1.59) |  | 1.38 (1.22-1.56) |
| Loneliness^d^ | 1.55 (1.23-1.95) |  |  | 1.44 (1.15-1.81) |  |  | 1.35 (1.07-1.70) |

^a^ Where available, updates of social isolation, loneliness, and covariates until age 77 years with data from Tromsø5 (2001), Tromsø6 (2007-08), and Tromsø 7 (2015-16).
^b^ Daily smoking, physical inactivity, low education, heavy alcohol consumption, high blood pressure, high cholesterol, and obesity.
^c^ Social isolation constructed from i) Living with a partner/spouse ii) participating in organized gatherings monthly or more frequent and iii) having friends to talk with and give support. Least isolated: Yes on all three (i-iii); modestly isolated: Yes on two out of the three; most isolated: Yes on zero or one of the three.
^d^ “Have you in the last two weeks felt lonely?” dichotomized to not lonely (no, a little) and lonely (a lot, very much). Not lonely reference.

Supplementary Table 6. Cox models with multiple imputation (m=10 imputed datasets and n=24126) and complete observations (n=17796). Hazard ratios, with 95% confidence intervals in parenthesis, of social isolation and loneliness in 25-69-year-old participants at baseline (Tromsø4). The Tromsø Study 1994-2023.

|  | Unadjusted | |  | Adjusted^a^ | |  |
| --- | --- | --- | --- | --- | --- | --- |
| Characteristic | Complete cases | Multiple imputation^b^ |  | Complete cases | Multiple imputation^b^ |  |
| Social isolation^c^ |  |  |  |  |  |  |
| Least isolated | 1 (reference) | 1 (reference) |  | 1 (reference) | 1 (reference) |  |
| Modestly isolated | 1.35 (1.25-1.46) | 1.32 (1.22-1.43) |  | 1.22 (1.13-1.31) | 1.21 (1.13-1.29) |  |
| Most isolated | 1.75 (1.59-1.92) | 1.68 (1.54-1.83) |  | 1.48 (1.35-1.63) | 1.45 (1.32-1.60) |  |
| Loneliness^d^ | 1.19 (1.00-1.41) | 1.28 (1.13-1.45) |  | 1.11 (0.94-1.32) | 1.16 (1.02-1.32) |  |

^a^ Daily smoking, physical inactivity, low education, heavy alcohol consumption, high blood pressure, high cholesterol, and obesity
^b^Logistic and multinomial logistic regression were used to impute. Results were combined with Rubin’s rule.
^c^ Social isolation constructed from i) Living with a partner/spouse ii) participating in organized gatherings monthly or more frequent and iii) having friends to talk with and give support. Least isolated: Yes on all three (i-iii); modestly isolated: Yes on two out of the three; most isolated: Yes on zero or one of the three.
^d^ “Have you in the last two weeks felt lonely?” dichotomized to not lonely (no, a little) and lonely (a lot, very much). Not lonely reference.
